# Criminal Abortion and the Public Press

**Published:** 1867-04

**Authors:** 


### Editorial Department.

### Criminal Abortion and the Public Press.

In our March number we mentioned, as an item to be noticed, that the *Northern Christian Advocate*, in an editorial article, covering more than an entire page of the sheet, had presented its readers in full, all the facts and consequences of criminal abortion. This is the first public discussion of the subject we have ever known, though the professional journals have for a long time been frequently occupied with the evil effects and crimes of preventing conception, and destroying the human embryo.* This newspaper expose of it, has so much truth, and with all, is so fear-

* Since the above article was written, we have received *The Congregationalist*, containing an article from Rev. John Todd, D. D., upon this subject. He says: "I appeal to New England women—the daughters of an ancestry who never were spotted with the blood of innocents, who never stifled the natural longings of a mother's heart and never quenched life immortal for the sake of ease or fashion and ask them if it is so, that they are so degenerated that they cannot meet the holiest position and less and outspoken, that we make the following extract, showing how it is regarded, what frightful ravages it has made, and how long one of the most revolting crimes in heathenism is practiced in modern churches, until now without protest, in religious papers:

"We expect to show that a species of infanticide is fearfully prevalent in American society—that the practice is murder—that it is a prolific and almost exclusive source of American female diseases—that it eclipses in iniquity, and is promotive of nearly all other social crimes, and that, therefore, the prevalent toleration and excuses for the practice are outrages against decency, humanity, and high Heaven.

The evil in question, though its widest prevalence has been attained with the past decade, already has a literature of which we name but fragments. And here we anticipate the suggestion that the discussion should be still left exclusively to medical men and publications. Unfortunately for all, such has been the case for ten years. All honor to the noble profession which has so faithfully uttered its warning, but, alas! the needed alarm, while it reaches but a tithe of the people, is also powerless to create that correct Christian sentiment which will throttle the monster, abuse and socially outlaw, the unnatural sinner against God.

The crime in question is *fœticide*, or the killing of unborn children. When necessary, it is simple 'abortion;' when unavoidable under certain circumstances, miscarriage, and when unnecessary, "Criminal Abortion"—Murder, as we will presently prove, and until then, assume.

Criminl abortion was, and is, equally wide-spread, and has not, as with us, originated during later years. Heathendom inherits barbarism; we have relapsed into it. Juvenal, born about A. D. 38, was by no means a prude, but the brutishness of Roman women stung him into protest. Near the close of his sixth, longest and most denunciatory satire, contrasting the poor and the rich, he says:

'Yet these, when circumstances so require, are ready to encounter the perils of childbirth, and endure all the irksome toils of nursing. But rarely does a gilded bed contain a woman lying-in; so potent are the arts and drugs of her that can ensure barrenness, and for bribes kill men while yet unborn.'

In Japan these 'drugs' are sold by priestly venders, and modern Egypt, like America, is cursed with gangs of quick abortionists and 'irregular practitioners' whose scoundrel 'arts kill men for bribes' and richly deserve the halter. Would to God the curse were confined to the Eastern continent. On this, heathen populations from Hudson's Bay to Peru, including several Indian tribes, practice fœticide. Alas! this species of murder is not restricted to the uncivilized, the unchristianized, or to those not enrolled within Christian churches. We have examined the subject somewhat closely for years and have been cruelly shocked by statistics and discoveries made, yet even, we are assured that only the observant, constant medical pracduties ever imposed on woman? We can show that France, with all her atheism, that Paris, with all her license, is not so guilty in this respect, as is staid New England, at the present hour." He has treated the subject in his unsurpassed style, and his article cannot but impress the thousand murderesses of New England churches and fashionable circles, with a sense of guilt and shame, with an idea that the curtain of secrecy has been lifted, and that the eye not of God only, but of the world, which they much more dread and fear, is upon them.

titioner can realize how widely and deeply this perversion of nature has demoralized, and is more widely and deeply demoralizing society.

The Protestant Church is to-day blackly stained by this crime of child-murder—and our Christianized society deserves the stinging satire of Juvenal. Let both be rebuked by Minitius Felix who, with others, wrote to show that Christ's followers are not like idolatrous heathen. Here is an extract addressed to the countrymen of Juvenal:

'Some of you will not give them (the children) liberty to be born, but by cruel potions procure abortion, and smother the hopeful beginnings of what would come to be a man.'

Miscarriages are frequent, and certain uninstructed or embruted feminine circles have their centers where the means employed and successes attained are topics of exchangable information. For instance, we could prove that in one little village of one thousand inhabitants, prominent women have been guilty of what we will presently show to be *murder*. And, sadder still, half of these are members of Christ's Church."

The above are quotations from the paper, not placed in their connections, but grouped, for the purpose of showing how truthfully, fearlessly and earnestly this subject has been presented.

There are some other subjects in near relation to this, which we should be glad to have treated as faithfully and earnestly by the same author. The newspapers have, for years, been the organs for innumerable quacks; not an issue, but contained the vilest, most barefaced, unscrupulous and infamous advertisements, which charlatans and mountebanks could invent. Christian newspapers even, have been the medium of communicating the most villainous of these productions, and we have sometimes turned in disgust and loathing from the perusal of the most popular of these sheets, with the conviction that they would be the means of destroying more lives, and ruining more souls, than they could be in any way instrumental in saving.

How comes it, that in every household is understood how to "avoid or destroy the legitimate fruits of matrimony?' It has been "attained with the last decade," because the public press has furnished the literature. The public papers have not hesitated to allow paying advertisers to furnish their own copy, and in gratuity given an editorial endorsement, or, if not quite that, have published local item advertisement, in such manner as to make the unsuspecting believe it was endorsed. This crime, so bitterly complained of by the *Northwestern Christian Advocate*, is the legitimate fruit of the *medical* column in our newspapers, both secular and religious; and we do not hesitate to charge back upon the religious and secular press participation in the crime, not only of child-murder, but of a great deal of manslaughter in the first degree. They have been familiarizing the women and children of this country, with the means not only of preventing offspring and destroying it while yet unborn, but have been carrying into families and communities untold evils, not only in worthless and injurious nostrums, but also in sensational advertisements, designed to operate upon the fears and excited imaginations of the young and inexperienced, causing them to believe that they were rapidly becoming the victims of incurable maladies, unless rescued by immediate application to the subscriber, who could send certain remedies, of never failing efficacy. Even the same paper containing the excellent article referred to, has a long advertisement, stealing the livery of the "Methodist Book Concern" in which to serve the devil.

Its commencement is so characteristic that we copy for the benefit of our readers, and to show the *Christian Advocate* how it appears outside its colums:

"*To the readers and patrons of the Northwestern Christian Advocate:*—I have been a reader and patron of the *Advocate* for several years, and am not unknown to the many readers of this long-established and widely circulated journal—not as a literary or scientific correspondent, but as a "regular" *contributor* to its advertising columns and the exchequer of the Methodist Book Concern. In this general way I have been introduced to its thousands of readers, many of whom have become my personal correspondents and friends. To all these I am well known as a citizen of Cincinnati, and as a medical specialist, who, for the last eighteen years, has been engaged in treating diseases of the Nose, Throat and Lungs by the inhalation of cool medicated air. Many hundreds of its readers have consulted me by letter and in person for professional advice."

After a long account of all sorts of diseases cured by his "cool medicated air" and of a book of one hundred pages which he proposes furnishing to all the subscribers of the *Northwestern Christian Advocate*, "*without charge if they wish it,*" he says, "The book cost me *twenty-five cents* a number. Those who desire and can pay for it, should send me that amount, as a simple act of justice," etc., etc., and closes his very long and absurd advertisement with the following:

"Dr. Reid, editor of the *Western Christian Advocate*, in noticing this book, through the columns of that journal, February 22, 1866, uses the following language: 'An honored friend and venerated minister has presented us with a copy of this work, with the respects of the author, and an earnest request on his own part that we shall recommend Dr. Wolfe and his remedies. Our brother assures us that the life of his own son has been saved by the treatment, and refers us to other well known friends likewise benefited.'"

Newspapers and those who edit and own them, are not without sin, and though this paper is remarkably free from objectionable notices, still it is perhaps accidental, and not from principle. The appearance of the article upon criminal abortion is very creditable to it, and we respect its editor for his outspoken and fearless denunciation of a great evil and sin. We have taken some pains to show some other abuses which he can help correct, and have no doubt but in due season he will do his part manfully. The time is coming—but we fear it is yet a great way off—when popular reading will be free from these abuses, and when imposters and quacks will not get ministers and editors to endorse and recommend them. We have sometimes wondered why Christian people, as a body, were more ready to believe in the marvellous, the inconsistent, and the inexplicable in medical matters, than others who were not more discriminating in many respects. We have never known anything in the teachings of Christianity which would naturally lead to unthinking belief in the inconsistencies of charlatans or the vagaries of quacks; which would make men more blind or more easily deluded, but we have often believed that they are so, and failed of satisfactory explanation. It is a reflection upon the intellectual acuteness of men, to be guilty of adopting error and adhering to it, when within reach of adequate and positive knowledge, especially if they manifest no desire, or even willingness to be acquainted with the grounds of a rational faith; and it is impossible for the educated and intelligent, to respect those within reach of the truth, who "bow down and worship wooden gods." When I inquire of my spiritual adviser, why is it that church-going people so frequently, in medical matters, "wander upon the dark mountains and perish," why worship, when the true God is known? he replies, "the worst of the delusion I believe is over; the heathen are to be forgiven because they do not know God." I accept the explanation, and believe when ministers and editors of Christian papers, desire to know the truth, and are not ready to promulgate error and absurdity, then the crimes and sins of which we complain, will be less frequent; or, if not, if the truth is plainly and fearlessly proclaimed, the poor, deluded victims cannot come up in the judgment and lay these sins to their charge.

